# Predictors of mortality in a cohort of tuberculosis/HIV co-infected patients in Southwest Ethiopia

**DOI:** 10.1186/s40249-016-0202-1

**Published:** 2016-12-05

**Authors:** Hailay Gesesew, Birtukan Tsehayneh, Desalegn Massa, Amanuel Gebremedhin, Hafte Kahsay, Lillian Mwanri

**Affiliations:** 1 Department of Epidemiology, College of Health Sciences, Jimma University, Jimma, Ethiopia; 2 Discipline of Public Health, Faculty of Medicine, Nursing and Health Sciences, Flinders University, Adelaide, Australia; 3 School of Statistics and Mathematics, Faculty of Science, University of Alberta, Edmonton, Canada; 4 Department of population and Family Health, College of Health Sciences, Jimma University, Jimma, Ethiopia; 5 ART Clinic, Filtu Hospital, Somali, Ethiopia

**Keywords:** Tuberculosis, HIV, Co-infection, Mortality, Survival, Retrospective cohort, Ethiopia

## Abstract

**Background:**

Tuberculosis/HIV co-infection is a bidirectional and synergistic combination of two very important pathogens in public health. To date, there have been limited clinical data regarding mortality rates among tuberculosis/HIV co-infected patients and the impact of antiretroviral therapy on clinical outcomes in Ethiopia. This study assessed the incidence and predictors of tuberculosis/HIV co-infection mortality in Southwest Ethiopia.

**Methods:**

A retrospective cohort study collated tuberculosis/HIV data from Jimma University Teaching Hospital for the period of September 2010 and August 2012. The data analysis used proportional hazards cox regression model at *P* value of ≤ 0.05 in the final model.

**Results:**

Fifty-five (20.2 %) patients died during the study period and 272 study participants contributed 3 082.7 person month observations. Factors including: being aged between 35–44 years (AHR = 2.9; 95 % *CI*: 1.08–7.6), being a female sex worker (AHR = 9.1; 95 % *CI*: 2.7–30.7), being bed ridden as functional status (AHR = 3.2; 95 % *CI*: 1.2–8.7), and being at World Health Organization HIV disease stages 2 (AHR = 0.2; 95 % *CI*: 0.06–0.5), 3(AHR = 0.3; 95 % *CI*: 0.1–0.8) and 4(AHR = 0.2; 95 % *CI*: 0.04–0.55) were significant predictors of mortality for tuberculosis/HIV co-infected patients.

**Conclusions:**

Contrary to our expectations, the World Health Organization (WHO) HIV disease stage 1 was found to be a significant predictor of mortality. Higher mortality rates were observed in WHO disease stage 1 patients compared to patients in stages 2, 3 and 4. The current study also confirmed and reaffirmed known significant predictors of the mortality for tuberculosis/HIV co-infected patients including being 35–44 years, being a female sex worker and being bed ridden functional status. The occurrence of high death rate among tuberculosis/HIV co-infected cases needs actions to reduce this poor outcome.

**Electronic supplementary material:**

The online version of this article (doi:10.1186/s40249-016-0202-1) contains supplementary material, which is available to authorized users.

## Multilingual abstract

Please see Additional file 1 for translation into the five official working languages of the United Nations.

## Background

Tuberculosis/HIV (TB/HIV) co-infection causes a serious bidirectional and synergistic combination of illness in which HIV promotes the progression of latent tuberculosis infection to disease, and tuberculosis (TB) accelerates the progression of HIV disease to poor prognosis including death [1]. In many circumstances, HIV has been described as the main reason for failure to meet TB control targets in high HIV settings, and TB is a major cause of death among people living with HIV in similar settings [2]. It is also recognised that individuals with TB/HIV co-infection are highly likely to develop active TB disease than individuals without HIV [3] According to the 2010 World Health Organization HIV disease stages (WHO) report on TB profiles for different countries, Ethiopia was classified as a high burden TB, a high burden HIV and a high burden Multi-drug resistance TB (MDR-TB) nation [3]. The prevalence of TB was estimated to be 394 (173–623) per 100 000 populations including those co-infected with HIV, and there were 152 030 new cases of which 3 190 were below 15 years of age [2].

Worldwide, TB remains one among the leading causes of death from an infectious agent. For example, in 2014, an estimated 9.6 million people developed TB, of which 1.2 million (12 %) were co-infected with HIV [4]. In 2015, 33 % of HIV deaths were due to TB showing that it is still a leading killer of HIV-positive people [5]. Different studies have indicated that TB is often the first manifestation of HIV infection, and it is the leading cause of death among HIV-infected patients in Africa [6, 7]. Globally, a third (33 %) of population living with HIV are infected with TB, and more than two third (70 %) of TB patients have been reported to live with HIV in Sub-Saharan Africa [3, 7]. In 2004, a study conducted in Ethiopia reported a TB/HIV co-infection prevalence of 18 % ranging from 8.3 % (in Silte zone) to 35.3 % (in South Omo zone) [8]. In 2015, according to the study conducted in south Ethiopia [9], 18.2 % of the HIV infected patients were found to have tuberculosis.

A wide range of factors that influence TB/HIV co-infection mortality have been reported. These include: age, gender, marital status, level of education, religion, occupation, residence, weight, AIDS staging, TB clinical presentation and calendar year [10, 11]. Previous studies have focused mainly on the survival rates of general HIV patients, giving less attention to TB/HIV co-infected patients [12–15]. A few studies in Ethiopia have analysed factors associated with mortality in HIV-infected TB patients [16, 17] and some studies [18, 19] took into account drug susceptibility patterns of Mycobacterium tuberculosis outside Ethiopia. However, these studies assessed the mortality rates of HIV positive and negative patients on anti-TB treatment. To date, there have been limited clinical data regarding mortality rates among TB/HIV co-infected patients and the impact of antiretroviral therapy (ART) on clinical outcomes in Ethiopia. The aim of the current study was to investigate the mortality rate of TB/HIV co-infected patients who developed TB after commencing ART in order to gain a better insight of the associated factors.

## Methods

The study was conducted in Jimma University Teaching Hospital (JUTH), 357 km southwest of Addis Ababa, the Capital City of Ethiopia. Jimma town is situated in Oromia region, a region that accounts for the highest number of HIV infected people in Ethiopia [20]. Jimma is also located near Gambella, a region that contributes the highest prevalence rate of HIV in Ethiopia [20]. A refugee camp located near the study setting zone brings in a large number of refugees from different African countries (particularly South Sudan), and the high emigration and immigration from and to Jimma places this town at a heightened risk of HIV and TB infection and transmission. Jimma teaching hospital is the largest health facility in Jimma town, its catchment area comprising a population of 2 486 155 inhabitants of which 89.69 % are rural dwellers [21]. The hospital caters for both Jimma and Gambella zones providing a substantial amount and important data which were favourable for this study.

A retrospective cohort study was conducted using JUTH records from September 1, 2010 to August 31 2012. The target population was all patients aged 15 years and above, who were HIV infected, on ART and developed TB when receiving the care from JUTH.

Death due to any cause was the event. Age, sex, religion, educational level, marital status, occupation, residence, number of people living in the household, accessibility to safe water, accessibility of electricity, number of bedrooms in the household, functional status, baseline CD4 cells count, drug regimen, WHO clinical disease stage, disclosure, risky sexual behaviour, tobacco smoking and alcohol drinking were the independent variables. WHO clinical disease staging was defined based on the current ART guideline for Ethiopia [22]. A risky sexual behaviour was dichotomized as ‘no’ (has regular sexual partner in the last three months) and ‘yes’ (has one or more casual sexual partners in the last three months). Condom use was also dichotomized as yes if ‘use rarely, sometimes, mostly or always during sexual intercourse’, and no if ‘never use during sexual intercourse’. Patients were considered as alcohol consumers if ‘they drink rarely, sometimes, mostly or always’ in their lifetime. Similarly, they were considered as cigarette smokers if ‘they smoke rarely, sometimes, mostly or always’ in lifetime.

Data extraction was performed between August and October 2013. A data extraction checklist (Annex I) was used to collect information from the patient cards, registration and log books. Data were entered in to Epi-data and then exported to SPSS version 22.0 for mackintosh for analysis. Incomplete records were excluded from analysis. The main end point in this study was death due to any cause among the TB/HIV co-infected patients. Individuals defaulted, lost to follow up, transferred out and survivors at the end of the study period were considered as censored. Finally, the out-come of each subject was dichotomized in to censored or event. The data were cleaned and edited before analysis. Data editing and exploration was undertaken to exclude odd codes or items that were illogical.

The analysis of both descriptive and inferential statistics was conducted. The cohort descriptive statistics included mean, median, standard deviations, and range values for continuous data; percentage, frequency tables, and graphs for categorical data. For the comparison of time to recovery among the different groups of patients, Kaplan Meir curve was used. Bivariate and multiple cox regression model with stepwise variable selection procedural was used to identify independent predictors of mortality. The assumption for proportional hazard was assessed graphically. Variables with *P*-value of <0.25 in the bivariate cox regression analysis were considered as candidate variables for multiple cox regression analysis. *P*-value of ≤5 % was considered significant in the final model.

## Results

### Baseline socio-demographic and economic characteristics of the study participants

Table 1 describes baseline socio-demographic and economic characteristics of the study participants. Two hundred eighty nine (289) TB/HIV co-infected patients were registered during September 1, 2010 and August 31, 2012. Complete records of 272 TB/HIV co-infected patients followed up for a mean of 340 days were included in the study. The mean age of the study participants was 32(±8.53) years with the majority of patients aged between 25–34 years. More than half (58.3 %) of the study participants were females. Muslims by religion and daily labourers by occupation respectively, represented 58.1 % and 31.6 % of participants. Almost half (51.5 %) and 60.7 % of participants respectively were formally educated and married. In terms of residence, almost 70 % of them were urban dwellers. A significant proportion (76.1 %) of participants had water and electricity in their residence and less than five people resided in the household. A significant number of participants lived in a single bedroomed (44.5 %) or two bedroomed (40.5 %) households.

**Table 1:** Baseline socio-demographic characteristics of Tb/HIV co-infected patients at JUTH, Southwest Ethiopia, 2013

Variable (*n* = 272)	Value	Number	Percent
Age (in years)	15–24	38	13.9
	25–34	140	51.5
	35–44	69	25.4
	> = 45	25	9.2
Sex	Male	114	41.9
	Female	158	58.1
Occupation	Government employed	48	17.7
	NGO*	46	16.9
	Farmer	80	29.4
	Daily labor	86	31.6
	Commercial Sex Worker	12	4.4
Educational status	Illiterate	81	29.8
	Read and write	51	18.8
	Formal education	140	51.4
Religion	Orthodox	76	27.9
	Muslim	134	49.3
	Protestant	50	18.4
	Catholic	12	4.4
Marital status	Married	165	60.7
	Single	58	21.3
	Divorced	36	13.2
	Widowed	13	4.8
Residence	Urban	189	69.5
	Rural	83	30.5
Number of people living with	<5	210	77.2
	> = 5	62	22.8
Water	Yes	207	76.1
	No	65	23.9
Electricity	Yes	207	76.1
	No	65	23.9
Room	1	121	44.5
	2	110	40.4
	3	31	11.4
	4	10	3.7

*as Non-governmental organizations

### Clinical and behavioural characteristics of the study participants

Table 2 presents clinical and behavioural characteristics of the study participants. In the cohort, 176 (64.7 %) participants had disclosed about their HIV status to their closest person such as their sexual partner or the next of kin. The majority of the patients were on first line ART regimen, working functional status and WHO stage 3. About 60 % of the study participants had low CD4 count (<200 cells/copies) and the median (range) CD4 count was 172.5 (425) cells/copies. When compared to other types of TB (extra pulmonary or mixed), pulmonary TB accounted for the majority (78.3 %) of cases. More than 85 % of patients had accessed the service care as new TB cases. Nearly 70 % of participants’ sexual partners were HIV positive and more than 70 % of study participants had risky behaviours. For example, four out of five patients (82.7 %) reported not using condoms to protect themselves from sexually transmissible diseases.

**Table 2:** Clinical and behavioural characteristics of Tb/HIV co-infected patients at JUTH, Southwest Ethiopia, 2013

Variable (*n* = 272)	Value	Number	Percent
Disclosure	Yes	176	64.7
	No	96	35.3
Function	Work	138	50.8
	Ambulatory	95	34.9
	Bed redden	39	14.3
WHO stage	1	17	6.3
	2	67	24.6
	3	124	45.6
	4	64	23.5
Regimen	First line	229	84.2
	Second line	43	15.8
CD4	<200 cells/copies	163	59.9
	> = 200 cells/copies	109	40.1
	Median (range)	172.5 (425)	-
TB type	Pulmonary	213	78.3
	Extra pulmonary	14	5.2
	Mixed	45	16.5
Mode of TB entry	New	232	85.3
	Relapse	27	9.9
	Dropout	13	4.8
Partner HIV status	Positive	189	69.5
	Negative	1	0.4
	Unknown	82	30.1
Risky behaviour	Yes	191	70.2
	No	81	29.8
Condom use	Yes	47	17.3
	No	225	82.7
Tobacco smoking	Yes	126	46.3
	No	146	53.7
Alcohol drinking	Yes	76	27.9
	No	196	72.1

### Mortality status of study participants

**Fig. 1:**
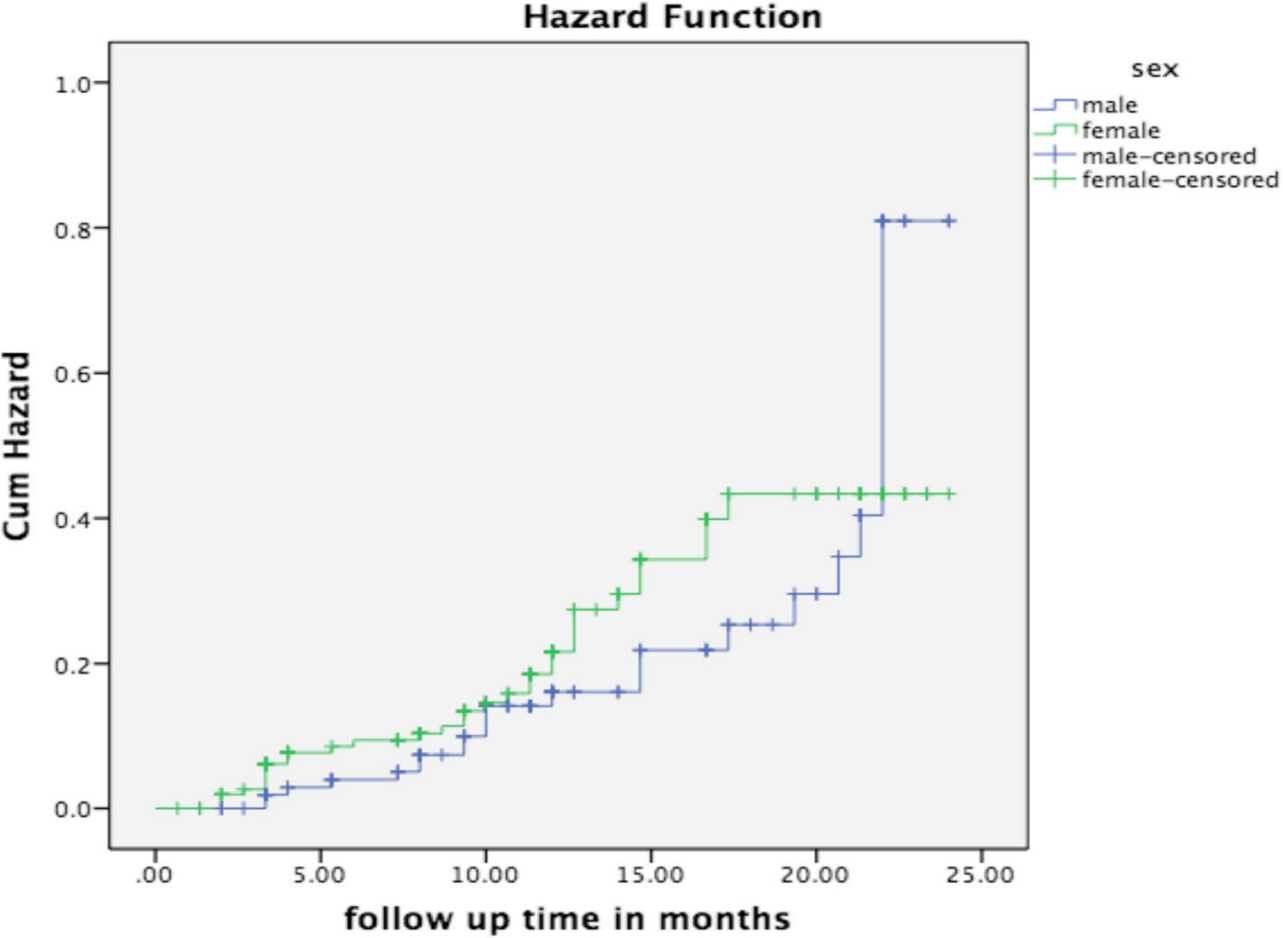
Mortality status by sex-This figure presents mortality status of the study participants by sex using Kaplan-Meier. It shows that females died more than males

**Fig. 2:**
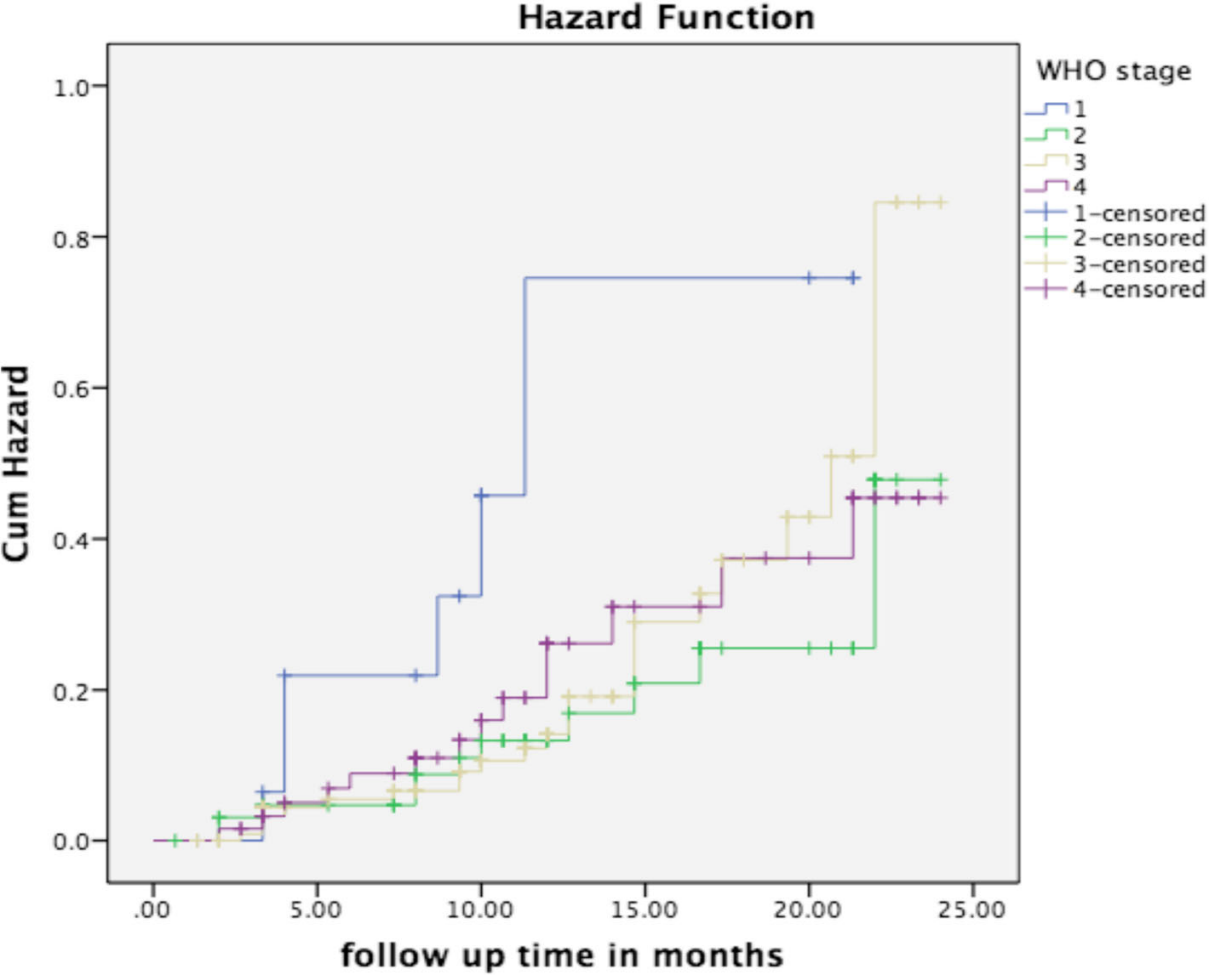
Mortality status by WHO stage- This figure presents mortality status of the study participants by WHO stage using Kaplan-Meier. It shows that TB/HIV co-infected patients diagnosed as WHO clinical stage 1 died more than WHO clinical stages 2, 3 and 4

**Fig. 3:**
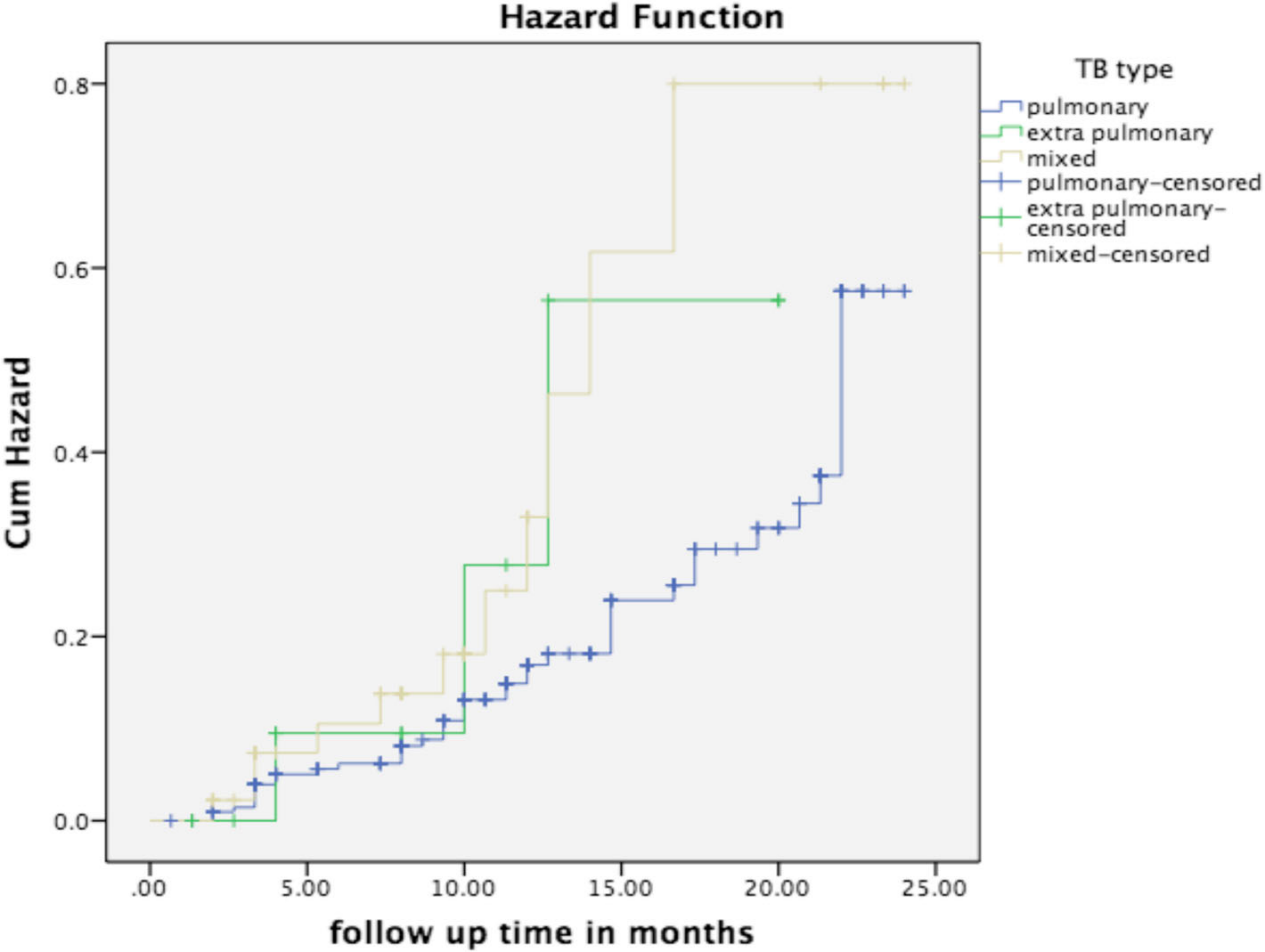
Mortality status by Tuberculosis type- This figure presents mortality status of the study participants by tuberculosis type using Kaplan-Meier. It shows that TB/HIV co-infected patients diagnosed as pulmonary tuberculosis died less than extra-pulmonary tuberculosis

Figures 1, 2 and 3 respectively present mortality status of study participants by sex, WHO stage and TB type using Kaplan-Meier graphs. A total of 55 (20.2 %) TB/HIV co-infected patients died during the study period. In addition, records of three, four and seven participants showed lost to follow up, defaulting, and transferred out respectively. The 272 study participants contributed to 3 082.7 person month observations (PMO) to the current study.

### Predictors of mortality in TB/HIV Co-infected patients during TB treatment

Table 3 presents predictors of mortality in TB/HIV Co-infected patients during TB/HIV treatment. Bivariate cox regression analysis showed that the risk of death was statistically different by age, educational status, occupation and WHO staging of HIV. After adjustment and checking the fitness of the model; being aged between 35–44 years, being a female sex worker (FSW), being a bed ridden functional status, and being WHO stages 2, 3 and 4 were independent predictors in the multiple Cox regression analysis.

**Table 3:** Multiple cox regression predictors of mortality among Tb/HIV patients at JUTH, Southwest Ethiopia, 2013

Variable	Tb/HIV Co-infection Status (*n* = 272)		Crude Hazard Ratio (95%*CI*)	Adjusted Hazard Ratio (95 % *CI*)
	Alive, *n* (%)	Died, *n* (%)		
Age in years				
15–24	32 (14.7) 114	6 (10.9)	1	1
25–34	(52.5) 48	26 (47.3) 21	1.1 (0.5–2.8)	1.4 (0.6–3.8)
35–44	(22.1)	(38.2)	2.2 (0.9–5.4)*	2.9 (1.08–7.6)*
> =45	23 (10.7)	2 (3.6)	0.6 (0.1–2.7)	0.3 (0.05–1.6)
Sex				
Male	91 (41.9)	23 (41.8)	1	1
Female	126 (58.1)	32 (58.2)	1.1 (0.7–1.9)	1.7 (0.8–3.5)
Educational status				
Illiterate	61 (28.1)	20 (36.4)	1	1
Read and write	39 (18)	12 (21.8)	0.7 (0.3–1.3)	0.9 (0.3–2.4)
Formal education	117 (53.9)	23 (41.8)	0.4 (0.2–0.7)*	0.6 (0.2–1.8)
Occupation				
Gov’t employed	40 (18.4)	8 (14.5)	1	1
NGO	37 (17.1)	9 (16.4)	1.1 (0.4–2.9)	0.9 (0.4–2.6)
Farmer	69 (31.8)	11 (20)	1.06 (0.4–2.6)	0.7 (0.3–1.9)
Daily labour	64 (29.5)	22 (40)	2.4 (1.04–5.3)*	2.1 (0.9–4.9)
CSW	7 (3.2)	5 (9.1)	6.7 (2.2–20.9)*	9.1 (2.7–30.7)*
Disclosure				
Yes	139 (64.1)	37 (67.3)	1	1
No	78 (35.9)	18 (32.7)	0.7 (0.4–1.1)	0.7 (0.3–1.8)
Function				
Work	112 (51.6)	26 (47.3)	1	1
Ambulatory	79 (36.4)	16 (29.1)	0.6 (0.3–1.09)	1.05 (0.5–2.1)
Bed ridden	26 (12)	13 (23.6)	0.5 (0.2–1.05)	3.2 (1.2–8.7)*
WHO stage				
1	11 (5.1)	7 (12.7)	1	1
2	55 (25.3)	12 (21.8)	0.3 (0.1–0.9)*	0.2 (0.06–0.5)*
3	101 (46.5)	23 (41.8)	0.4 (0.2–1.02)	0.3 (0.1–0.8)*
4	50 (23.1)	13 (23.7)	0.4 (0.2–1.09)	0.2 (0.04–0.5)*
Tb type				
Pulmonary	172 (79.3)	41 (74.5)	1	1
Extra-pulmonary	11 (5.1)	3 (5.5)	0.6(0.3–1.07)	2.6(0.5–12.2)
Mixed	34 (15.6)	11 (20)	1(0.3–3.6)	0.9(0.3–2.7)
Risky behaviour				
Yes	153 (70.5)	38 (69.1)	1	1
No	64 (29.5)	17 (30.9)	0.9 (0.5–1.6)	0.8 (0.4–1.9)
Partner HIV status				
Positive	150 (69.1)	39 (70.9)	1	1
Negative/Unknown	67 (30.9)	16 (29.1)	1.4 (0.8–2.5)	0.5 (0.2–1.5)

*Statistically significant at *P* < 0.05

## Discussion

The current study indicates the occurrence of a significant mortality rate (20.2 %) amongst TB/HIV co-infected patients in JUTH. However, this finding was not dissimilar to the WHO reports that have shown TB’s contribution of up to 26 % of HIV mortality elsewhere [23]. Similar findings have also been echoed by studies in Ethiopia and Malaysia [16, 24].

Study participants whose ages were between 35–44 years old had nearly 3 times higher risk of (AHR = 2.9, 95 % *CI*: 1.08–7.6) death than those below 25 years old, the finding which is similar with a study conducted in Brazil [10]. Some attribution to this occurrence could be that older patients are more likely to be diagnosed with HIV and/or TB late. It is well acknowledged that late diagnosis facilitates poor prognosis and deaths due to immune deficiency from rapid progression to Acquired Immune Deficiency Syndrome (AIDS) and extra-pulmonary tuberculosis [25–28]. This has implications for practice indicating the heightened need for early diagnosis and treatment of both HIV and TB. Similar to studies elsewhere [29], the odds of TB/HIV death was 9 times for CSW compared to other occupation such as government employees (AHR = 9.1, 95 % *CI*: 2.7–30.7). This finding is significantly important for nations health systems, and may have policy and practice implications in Ethiopia and elsewhere, including prioritizing this group as a key population and addressing their needs and designing more effectively targeted management.

Regarding functional status, bedridden patients had three times (AHR =3.2, 95 % *CI*: 1.2–8.7) increased risk of mortality than patients recorded being in working status. This finding was in agreement with another study conducted in Bahir Dar, Ethiopia [16], where bedridden patients were reported to be nearly 4 times at risk of dying than patients in work functional status category (AHR = 3.88; 95%*CI*: 2.15–7.02). These findings do not come as a surprise because patients who are bedridden have probably been exposed to more infections and have poorer health outcome with vicious cycle of poorer immunity, which can lead to patients easily succumbing to opportunistic infections and death [30]. It is thus plausible to recommend mandatory and frequent screening for opportunistic infections and other diseases in order to institute early and effective management strategies to reduce preventable mortalities.

Similar to the study conducted in Bahir Dar [16], the Kaplan-Meier graph on mortality status of TB/HIV co-infection by TB type, indicated that extra pulmonary TB patients in the current study died more than pulmonary TB patients. However, the findings of the current study were not statistically significant in the final model of the cox regression and we hypothesise that a small sample size could attribute to this effect.

Patients in WHO stages 2, 3 and 4 compared to stage 1 were found to have lower mortality risk. For example, the risk of dying in patients diagnosed as WHO HIV stage 3 was decreased by 70 % (AHR = 0.3; 95 % *CI*: 0.1–0.8) compared to WHO stage 1 patients. This is surprisingly uncommon, and contrasts findings from similar studies [16, 30, 31]. We hypothesise that these differences could be a result of paradoxical and unmasking types of TB-associated immune reconstitution inflammatory syndrome (TB-IRIS) [32]. IRIS is a clinical debilitating condition which occurs after the commencement of ARTs due to inflammatory responses against pathogens and presents with high fevers, distress, lymphadenopathy, meningitis, central nervous system lesions, ascites and radiological worsening of pulmonary infiltrates [33–35]. We thus argue that, when the patient passes the IRIS stage, it is likely to live longer than a patient at stage 1 who does not survive IRIS. However, this issue is a new and unique finding and warrants further research and exploration.

The major limitations of the current study were data incompleteness and small sample size. In addition, variables that could potentially have confounding effects such as alcohol abuse, income, illicit drug use, drug resistance, co-morbidities, treatment adherence and severity of immune suppression were not measured. Due to limitations in finance and human resources, the outcome status of defaulted, lost to follow-up and transferred out patients was not traced. This could have underestimated the incidence of death and subsequently affect the mortality estimates.

## Conclusions

The findings of the current study have informed new unexpected predictor of mortality: higher mortality rate in WHO HIV stage 1 compared to WHO HIV disease stages 2, 3 and 4. It has also reaffirmed known significant predictors of the mortality for TB/HIV co-infected patients including being 35–44 years, being a CSW and being at bed ridden functional status. The occurrence of high death rates among TB/HIV co-infected cases needs actions to reduce this poor outcome. The findings of the current study have policy and practice implications and they call for effective management strategies for TB/HIV co-infection including improved availability of early diagnosis and improved availability of ARVs to reduce this high death rates. Further studies are recommended with larger sample size in order to obtain comprehensive predictors of TB/HIV co-infection deaths.

## Additional files

Additional file 1: Multilingual abstract in the five official working languages of the United Nation. (PDF 782 kb)
Additional file 2: Clinical and non-clinical data of Tb/HIV co-infectd patients attending in JUTH, Southwest Ethiopia. (SAV 17 kb)

## Abbreviations

AHR: Adjusted Hazard Ratio
AIDS: Acquired immuno deficiency syndrome
ART: Antiretroviral therapy
FSW: Female sex worker
HIV: Human immunodeficiency virus
IRIS: Immune reconstitution inflammatory syndrome
JUTH: Jimma University Teaching Hospital
MDR: Multi drug resistance
PMO: Person month observation
TB: Tuberculosis
WHO: World Health Organization
