# Supplementary material for: Predictors of mortality in a cohort of tuberculosis/HIV co-infected patients in Southwest Ethiopia
Source: Infect Dis Poverty. 2016 Dec 5;5:109. doi: 10.1186/s40249-016-0202-1 (PMC5137204; doi:10.1186/s40249-016-0202-1)
Supplement: Additional file 1: — Multilingual abstract in the five official working languages of the United Nation. (PDF 782 kb) [file 40249_2016_202_MOESM1_ESM.pdf]

Translation of the abstract into the five official working languages of the United Nations

تنبؤات الوفيات في مجموعة من المرضى المصابين مشاركة بالسل/وفيروس نقص المناعة البشرية في جنوب غرب إثيوبيا

هيلي جيسيسو، بيرتوكان تسيهينه، ديسالين ماسا، أمانويل جبريمدهين، هافت كاهساي، ليليان موانري

الملخص

الخلفية: الإصابة المشتركة بالسل/وفيروس نقص المناعة البشرية هي مزيج ثنائي الاتجاه والتآزر لاثنين من مسببات الأمراض الهامة جداً في مجال الصحة العامة. وحتى الآن، هناك معطيات سريرية محدودة بشأن معدلات وفيات المرضى المصابين مشاركة بالسل/وفيروس نقص المناعة البشرية وتأثير العلاج المضاد للفيروسات على النتائج السريرية في إثيوبيا. تُقَيِّم هذه الدراسة حدوث وتنبؤات وفيات الإصابة المشتركة بالسل/وفيروس نقص المناعة البشرية في جنوب غرب إثيوبيا .

الطرق: قارنت دراسة استعادية مجموعة بيانات السل/ وفيروس نقص المناعة البشرية من المستشفى التعليمي لجامعة جيما في الفترة من سبتمبر 2010 وأغسطس 2012. استخدم تحليل البيانات نموذج انحدار كوكس لمخاطر النسبية بالقيمة P لـ ≤ 0.05 في النموذج النهائي.

النتائج: توفي 55 (20.2٪) مريض خلال فترة الدراسة وساهم 272 مشارك في الدراسة بملاحظات 3082.7 شخص في الشهر. العوامل التي شملت: أن تكون الأعمار تتراوح بين 44-35 عاماً (AHR = 2.9، 95% فاصل ثقة: 7.6-1.08)، وتكون عاملة مؤنثة (AHR = 9.1، 95% فاصل ثقة: 30.7-2.7)، وطريحة الفراش كوضع وظيفي (AHR = 3.2، 95% فاصل ثقة: 1.2-8.7)، وكونها مريضة بفيروس نقص المناعة البشرية حسب منظمة الصحة العالمية في مراحل 2 (AHR = 0.2، 95% فاصل ثقة: 0.5-0.06)، 3 (AHR = 0.3، 95% فاصل ثقة: 0.8-0.1) و 4 (AHR = 0.2، 95% فاصل ثقة: 0.55-0.04) كانت مؤشرات هامة لوفيات المرضى المصابين مشاركة بالسل/وفيروس نقص المناعة البشرية.

الاستنتاجات: على عكس توقعاتنا، وُجِدَ أن مرحلة المرض1 بفيروس نقص المناعة البشرية حسب منظمة الصحة العالمية (WHO) تكون مؤشراً كبيراً للوفيات. ولوحظت معدلات وفيات أعلى للمرضى في مرحلة المرض 1 حسب منظمة الصحة العالمية مقارنة بالمرضى في المراحل 2 و 3 و 4. كما أكدت الدراسة الحالية وجددت التنبؤات الهامة المعروفة لوفيات المرضى المصابين مشاركة بالسل/وفيروس نقص المناعة البشرية التي تشمل كونهم بين 35-44 عاماً، وكونهن عاملات إناث، وكون الوضع الوظيفي طريحة الفراش. إن حدوث ارتفاع معدل الوفيات بين حالات المصابين مشاركة بالسل/وفيروس نقص المناعة البشرية يحتاج إجراءات للحد من هذه النتيجة السيئة.

Translated from English version into Arabic by Free bird, through

TRANSLATORS
WITHOUT BORDERS

埃塞俄比亚西南地区结核病-HIV 联合感染病人队列研究中预测死亡率的因素

Hailay Gesesew, Birtukan Tsehayneh, Desalegn Massa, Amanuel Gebremedhin, Hafte Kahsay, Lillian Mwanri

摘要：

引言：结核病与艾滋病合并感染是由公共卫生领域中非常重要的且能够交互协同作用的两种病原体所导致的。迄今，埃塞俄比亚结核病-艾滋病合并感染患者的死亡率和抗逆转录治疗的临床数据记录很少。本研究主要评价埃塞俄比亚西南部结核病与艾滋病合并感染的发病

率及死亡率的预测因子。
方法：本研究对 2010 年 9 月-2012 年 8 月吉马大学教学医院的结核病和艾滋病的病例数据信息进行了回顾性队列研究。采用了 COX 回归风险比例模型（*P*≤0.05）进行数据分析。
结果：研究期间共 55（占 20.2%）例死亡病例，272 例患者总计贡献了 3 082.7 人月的生存时间。年龄 35-44 岁（AHR=2.9；95%*CI*：1.08-7.6），女性性工作者(AHR=9.1；95%*CI*:2.7-30.7)，长期卧床（AHR=3.2；95%*CI*：1.2-8.7），以及世界卫生组织（WHO）艾滋病 2 期(AHR=0.2；95%*CI*：0.06-0.5)，3 期(AHR=0.3；95%*CI*：0.1-0.8)和 4 期(AHR=0.2；95%*CI*：0.04-0.55)等是结核病艾滋病合并感染患者死亡率的重要预测因子。
结论：与我们的预期相反，世界卫生组织艾滋病感染 1 期对病死率有显著的预测作用。世界卫生组织艾滋病 1 期的病死率明显高于艾滋病 2 期、3 期和 4 期。本研究再次明确了影响结核病艾滋病合并感染死亡率的一些已知的重要预测因子，其中包括年龄 35-44 岁，女性性工作从业者和长期卧床。我们需要采取一些相关措施来降低结核病艾滋病合并感染患者的高病死率。

Translated from English version into Chinese by Lu-Lu Huang, edited by Pin Yang

Prédicteurs de mortalité au sein d'une cohorte de patients co-infectés par la tuberculose et le VIH dans le Sud-Ouest de l'Éthiopie

Hailay Gesesew, Birtukan Tsehayneh, Desalegn Massa, Amanuel Gebremedhin, Hafte Kahsay, Lillian Mwanri

Résumé

Contexte : la co-infection par la tuberculose et le VIH est une combinaison bidirectionnelle et synergétique de deux très importants agents pathogènes dans le domaine de la santé publique. Il n'existait jusqu'à présent que peu de données cliniques relatives aux taux de mortalité parmi les patients co-infectés par la tuberculose et le VIH et l'impact du traitement antirétroviral sur les résultats cliniques en Éthiopie. La présente étude a évalué l'incidence et les prédicteurs de la mortalité due à une co-infection tuberculose/VIH dans le Sud-Ouest de l'Éthiopie.
Méthodes : une étude rétrospective de cohortes a consisté à regrouper des données sur la co-infection tuberculose/VIH de l'Hôpital universitaire Jimma pendant une période allant de septembre 2010 à août 2012. L'analyse de données a consisté à utiliser un modèle de régression à risques proportionnels de Cox à une valeur *P* de ≤ 0,05 dans le modèle final.
Résultats : 55 (20,2 %) patients sont décédés au cours de la période d'étude et 272 participants à l'étude ont contribué à hauteur de 3082,7 mois-personnes d'observation. Les facteurs étaient les suivants : être âgé de 35 à 44 ans (AHR=2,9 ; *IC* à 95 % : 1,08-7,6), être une prostituée (AHR=9,1 ; *IC* à 95 % : 2,7-30,7), présenter un statut d'alitement (AHR=3,2 ; *IC* à 95 % : 1,2-8,7) et présenter les stades de maladie suivants selon l'Organisation Mondiale de la Santé 2 (AHR=0,2 ; *IC* à 95 % : 0,06-0,5), 3 (AHR=0,3 ; *IC* à 95 % : 0,1-0,8) et 4 (AHR=0,2 ; *IC* à 95 % : 0,04-0,55). Il s'agissait de prédicteurs significatifs de mortalité pour les patients co-infectés par la tuberculose/VIH.

Conclusions : contrairement à nos attentes, le stade 1 du VIH de l’Organisation Mondiale de la Santé (OMS) s’est avéré être un prédicteur significatif de la mortalité. Des taux élevés de mortalité ont été observés chez les patients atteints de la maladie au stade 1 de l’OMS par rapport aux patients aux stades 2, 3 et 4. L’étude actuelle a aussi confirmé et réaffirmé des prédicteurs significatifs connus de la mortalité des patients co-infectés par la tuberculose/VIH, notamment être âgé de 35 à 44 ans, être une prostituée et présenter un statut d’alitement. La survenue d’un taux de décès élevé parmi les cas de co-infection par la tuberculose/VIH requiert la prise de mesures afin de réduire ce résultat insuffisant.

Translated from English version into French by eric ragu, through

Прогнозы о смертности туберкулезных/Вич-инфицированных пациентов на юго-западе Эфиопии.

Hailay Gesesew, Birtukan Tsehayneh, Desalegn Massa, Amanuel Gebremedhin, Hafte Kahsay, Lillian Mwanri

Реферат

Фон: Туберкулез/Вич-инфиция представляет собой двунаправленную и синергетическую комбинацию из двух важных возбудителей общественного здравоохранения. На сегодняшний день, мало клинических данных о смертности туберкулезных/Вич-инфицированных пациентов и влиянии антиретровирусной терапии на клинические исходы. Данная работа оценила заболеваемость и прогнозы туберкулезной/Вич-инфицированной смертности на юго-западе Эфиопии.

Методы: С помощью ретроспективного когортного исследования собирать туберкулезные/Вич-инфицированные данные из Jimma университетской больницы с сентября 2010 г. по август 2012 г.. По модели пропорционального риска кокса регрессии (P ≤ 0.05) в окончательном режиме.

Результаты: 55 (20.2%) пациентов умерли в течение периода исследования и 272 пациента посвятили 3082.7 человек-месяц наблюдений. Включают в важные прогнозы смертности туберкулезных/Вич-инфицированных пациентов такие факторы: будучи в возрасте 35-44 лет (AHR=2.9; 95%*CI*: 1.08-7.6), будучи женского пола работника (AHR=9.1; 95%*CI*: 2.7-30.7), будучи долгое время ложиться в кровать (AHR=3.2; 95%*CI*: 1.2-8.7), будучи на стадии 2 (AHR=0.2; 95%*CI*: 0.06-0.5), 3(AHR=0.3; 95%*CI*: 0.1-0.8) и 4(AHR=0.2; 95%*CI*: 0.04-0.55) Всемирной организации здравоохранения ВИЧ-инфекции.

Заключение: Вопреки нашим ожиданиям, стадия 1 всемирной организации здравоохранения ВИЧ-инфекции была определена как важный прогноз смертности. По сравнению со ставкой смертности пациентов, которые находятся на стадии 2, 3 и 4, наблюдается более высокая смертности для пациентов, находящихся на стадии 1 болезни WHO. Настоящими

исследованиями были подтверждены известные важные прогнозы смертности для туберкулезных/Вич-инфицированных пациентов, в том числе и пациентам 35-44 года, женского пола работник и пациенты долгое время ложутся в кровать. Актуально нужно принимать меры снизить смертность туберкулезных/Вич-инфицированных пациентов.

Translated from English version into Russian by Hao-Qi Zhang

Indicadores de Mortalidad en un grupo de pacientes co-infectados por tuberculosis y VIH en el sudoeste de Etiopía

Hailay Gesesew, Birtukan Tsehayneh, Desalegn Massa, Amanuel Gebremedhin, Hafte Kahsay, Lillian Mwanri

Resumen

Antecedentes: La co-infección por tuberculosis/VIH es una combinación bidireccional y sinergística de dos patógenos sumamente importantes en salud pública. Hasta la fecha, existe limitada información clínica en cuanto a los índices de mortalidad entre pacientes co-infectados por tuberculosis y VIH y el impacto de la terapia antiretroviral en los resultados clínicos en Etiopía. Este estudio evaluó la incidencia y los indicadores de mortalidad por co-infección por tuberculosis y VIH en el sudoeste de Etiopía.

Métodos: Un estudio de cohorte retrospectivo recabó información sobre tuberculosis/VIH del Teaching Hospital de la Universidad de Jimma para el período entre septiembre de 2010 y agosto de 2012. El análisis de datos utilizó el modelo riesgo proporcional de Cox a un valor *P* de ≤ 0.05 en el modelo final.

Resultados: 55 (20,2%) de los pacientes murieron durante el período del estudio y 272 participantes en el estudio contribuyeron 3 082.7 observaciones persona mes. Los factores incluyeron: tener entre 35 y 44 años de edad (AHR=2,9; 95%*IC*: 1.08-7.6), ser trabajadora del sexo (AHR=9,1; 95%*IC*: 2.7-30.7), el estar postrado en la cama como estado funcional (AHR=3,2; 95%*IC*: 1.2-8.7) y estar en la etapa 2 de enfermedad por VIH de acuerdo a la clasificación de la Organización Mundial de la Salud (AHR=0,2; 95%*IC*: 0,06-0,5), etapa 3(AHR=0,3; 95%IC: 0,1-0,8) y etapa 4(AHR=0,2; 95%*IC*: 0,04-0,55) fueron indicadores significativos de mortalidad para pacientes co-infectados por tuberculosis y VIH.

Conclusiones: Contrario a nuestras expectativas, se encontró que la etapa 1 de la enfermedad por VIH de acuerdo a la Organización Mundial de la Salud era un indicador significativo de mortalidad. Se observaron elevados índices de mortalidad en la etapa OMS 1 de la enfermedad en comparación con las etapas 2, 3 y 4. El estudio actual también confirmó y reafirmó indicadores de mortalidad conocidos y significativos para pacientes co-infectados por tuberculosis y VIH, incluidos el tener entre 35 y 44 años de edad, ser trabajadora del sexo y estar postrado en la cama como estado funcional. La ocurrencia del alto índice de mortalidad entre los casos co-infectados por tuberculosis y VIH necesita de acciones para disminuir estos resultados desfavorables.

Translated from English version into Spanish by Maria Alejandra Aguada, through

TRANSLATORS
WITHOUT BORDERS
